# Use of sawdust for production of ligninolytic enzymes by white-rot fungi and pharmaceutical removal

**DOI:** 10.1007/s00449-024-02976-8

**Published:** 2024-03-14

**Authors:** M. Hultberg, O. Golovko

**Affiliations:** 1 Department of Biosystems and Technology, Swedish University of Agricultural Sciences, Växtskyddsvägen 3, 234 56 Alnarp, Sweden; 2 Department of Aquatic Sciences and Assessment, Swedish University of Agricultural Sciences (SLU), 750 07, Uppsala, Sweden

**Keywords:** *Ganoderma lucidum*, Laccase, Mycoremediation, *Pleurotus ostreatus*, *Trametes versicolor*

## Abstract

Use of white-rot fungi for enzyme-based bioremediation of wastewater is of high interest. These fungi produce considerable amounts of extracellular ligninolytic enzymes during solid-state fermentation on lignocellulosic materials such as straw and sawdust. We used pure sawdust colonized by *Pleurotus ostreatus*, *Trametes versicolor*, and *Ganoderma lucidum* for extraction of ligninolytic enzymes in aqueous suspension. Crude enzyme suspensions of the three fungi, with laccase activity range 12–43 U/L and manganese peroxidase activity range 5–55 U/L, were evaluated for degradation of 11 selected pharmaceuticals spiked at environmentally relevant concentrations. Sulfamethoxazole was removed significantly in all treatments. The crude enzyme suspension from *P. ostreatus* achieved degradation of wider range of pharmaceuticals when the enzyme activity was increased. Brief homogenization of the colonized sawdust was also observed to be favorable, resulting in significant reductions after a short exposure of 5 min. The highest reduction was observed for sulfamethoxazole which was reduced by 84% compared to an autoclaved control without enzyme activity and for trimethoprim which was reduced by 60%. The compounds metoprolol, lidocaine, and venlafaxine were reduced by approximately 30% compared to the control. Overall, this study confirmed the potential of low-cost lignocellulosic material as a substrate for production of enzymes from white-rot fungi. However, monitoring over time in bioreactors revealed a rapid decrease in enzymatic ligninolytic activity.

## Introduction

Many anthropogenic chemicals used in everyday life, such as pharmaceuticals, are designed to have a biological effect in low concentrations. In addition, several of these compounds are persistent and end up in wastewater. Conventional wastewater treatment plants (WWTPs) can remove these compounds to varying degrees depending on the chemical properties of the compound and processes at the WWTP [1]. Residual compounds are spread into the environment, with potential impacts on ecological status and with risks of accumulation in the food chain.

Different approaches for increasing removal of pharmaceuticals at WWTPs are emerging. Currently, advanced technologies such as ozonation and adsorption on active carbon are being explored for large-scale treatment [2]. Considering the different needs of WWTPs and wide array of contaminating compounds, access to a range of treatment techniques will likely be required in the future. Bioremediation, i.e., use of biological systems for treatment of contaminants, has received considerable attention in recent decades [3]. This can partly be explained by the potential energy-saving and environmental compatibility of biological systems, but also by their high efficiency when the contaminants are present in low concentrations. Within available bioremediation approaches, enzyme-based bioremediation is currently the main focus for wastewater treatment [4, 5].

Enzyme-based bioremediation processes can employ different types of organisms, including plants, algae and microorganisms, but a particularly promising group is white-rot fungi [4], a blanket term for a group of fungal organisms that can efficiently degrade lignin, leaving a whitish film on the decayed wood. Important extracellular ligninolytic enzymes produced by white-rot fungi are laccases and peroxidases. These enzymes, classified as oxidoreductases, have non-specific substrate interaction and are well known for their capacity to degrade persistent contaminants [6, 7].

However, water is not a preferred habitat for white-rot fungi, which play a natural role as wood degraders in terrestrial ecosystems [8]. Several species of white-rot fungi are produced commercially on a large scale, and thus well-established technologies for their production are in place [9]. Fungal growth and extracellular production of ligninolytic enzymes take place in solid-state fermentation on lignocellulosic materials such as straw and sawdust. For edible white-rot species, such as *Pleurotus ostreatus* (oyster mushroom), lignocellulosic material colonized by the fungi is readily available, since an estimated 3–5 kg of spent substrate are generated per kg of mushrooms produced [10]. However, production of extracellular enzymes by white-rot fungi varies over time within the fungal growing cycle, with several studies suggesting that the highest enzymatic activity is observed in the early colonization phase [11, 12]. Thus, spent mushroom substrate might not be the best choice for production of white-rot fungi for bioremediation purposes and, instead, potentially lignocellulosic material colonized with white-rot fungi should be developed directly for use in water treatment.

The present study explored use of a low-cost substrate, sawdust, for production of laccase and manganese peroxidase by white-rot fungi. The impact of crude enzyme suspensions in reducing the concentrations of 11 different pharmaceuticals, commonly detected in wastewater [13], was then evaluated. Parallel, bioreactors packed with sawdust, colonized with the white-rot fungi *P. ostreatus*, were monitored in order to gain information about enzymatic activity over time.

## Materials and methods

### Microorganisms and their cultivation

Three white-rot fungi, *Pleurotus ostreatus* M2191 (oyster mushroom), *Trametes versicolor* M9911 (turkey tail mushroom) and *Ganoderma lucidum* M9726 (reishi mushroom) were used in the experiments. Grain spawn of these fungal strains was obtained from Mycelia BVBA, Belgium, and inoculated in the substrate, sawdust of birch (*Betula* sp., particle size 2–4 mm). No amendments, such as wheat bran to increase nitrogen content, were added to the sawdust. An amount of 210 g of sawdust (dry weight, dw) was used for each replicate and the moisture content of the substrate was set to 65% by addition of distilled water. The substrate was pasteurized at 65°C for 8 h and, after cooling, spawn was added to the substrate in a concentration of 10% of dw. The inoculated substrates were incubated for 14 days or used directly in the experiments. During incubation, the inoculated substrates were packed in boxes suitable for mushroom production (TP1600 #30 WH, Sac O2, Nevele, Belgium) and kept in a climate chamber at 22ºC.

### Experimental set-up: enzyme activity

Laccase activity and manganese peroxidase activity were determined after 14 days of incubation of inoculated sawdust substrate. First, 200 g of colonized sawdust (wet weight, ww) was soaked in 1 L of sterile distilled water and incubated at an orbital shaker (100 rpm) for 15 min at room temperature. The sawdust was then filtered off using a nylon filter (mesh size 1 mm) and enzyme activity was determined in the filtrate. For *P. ostreatus*, enzyme activity of the substrate was also determined after brief homogenization (15 s) of the colonized substrate in a Waring blender at low speed and filtering through a nylon filter with mesh size 0.1 mm. The enzyme activity was determined directly after homogenization and filtering.

Enzyme activity in water over time was determined in small-scale cylindrical Plexiglass (PMMA) bioreactors (diameter 4.5 cm, height 18 cm) with PVC lids. Vertical water flow at different rates (1 or 0.3 L/h) was applied using a peristaltic pump (IKS Vario blue II) connected to the top of each reactor. The reactors were designed to allow aeration, so that an air pump (50 L/h) and a diffuser could be connected. A 100 g (ww) portion of sawdust substrate colonized by *P. ostreatus* was added to each reactor, which resulted in substrate height of 15 cm. Thus, the volume of the reactor occupied by colonized sawdust was 0.24 L. Water was pumped through the substrate and laccase activity and manganese peroxidase activity in the outgoing water were determined as described below. Four different treatments (Exp. A–D), involving different colonization times, aeration and flow rates were applied using this set-up (Table 1).

**Table 1:** Conditions applied in the different bioreactor experiments (Exp.) on substrate colonized by white-rot fungi *Pleurotus ostreatus*

Conditions	Exp. A	Exp. B	Exp. C	Exp. D
Substrate	Sawdust	Sawdust	Sawdust	Sawdust
Fungal colonization time	14 days	14 days	0–12 days	14 days
Duration of experiment	24 h	24 h	10 days	10 days
Flow rate	1 L/h	1 L/h	0.3 L/h	0.3 L/h
Aeration	−	+	−	−
Water type	Tap	Tap	Tap	Tap

Laccase activity and manganese peroxidase activity were determined colorimetrically by detecting the oxidation product 2,6-dimethoxyphenol (DMP, *ε*_468_ = 49,600 M^−1^ cm^−1^), as described by Parenti et al. [14]. The reaction mixture contained 0.45 mL of sample and 0.5 mL of 10 mM DMP in 100 mM acetate buffer (pH 5). Absorbance was measured at 468 nm and one unit (U) of enzyme activity was defined as formation of 1 µmol of product per min. Manganese peroxidase activity was initiated by addition of MnSO_4_ (1 mM) and H_2_O_2_ (0.4 mM) as described by Field et al. [15], with peroxidase activity corrected for background laccase activity.

### Experimental set-up: removal of pharmaceuticals

In a first experiment, whole sawdust on which the selected white-rot fungi had been grown for 14 days were used for extraction of the enzymes. These substrates were soaked in sterile distilled water (200 g ww/L) for 2 h and the sawdust was filtered off with a nylon filter with mesh size 1 mm. Enzyme activity was determined and the samples were then directly spiked with a mixture of pharmaceuticals (described below). As controls, autoclaved samples of the suspension from *P. ostreatus* were used, with lack of enzyme confirmed by testing after autoclaving. A slight increase in the pH of the crude enzyme suspension, from 4.7 ± 0.1 to 5.1 ± 0.1, was observed after autoclaving, so the pH in the control treatment was corrected to 4.6 with the use of 0.1 M HCl.

In a second experiment, substrate colonized by *P. ostreatus* soaked in sterile distilled water (400 g ww/L) for 2 h was used. In this experiment, an additional treatment based on suspension of mixed sawdust was included and performed parallel with the treatment based on whole sawdust. For mixing, the substrate was subjected to brief homogenization (15 s) in a Waring blender at low speed. All suspensions were filtered through a nylon filter with mesh size 0.1 mm. Enzyme activity was determined and the samples were then directly spiked with a mixture of pharmaceutical (described below). The control was autoclaved samples of the suspension from the mixed *P. ostreatus* substrate, with pH corrected to 4.6 using 0.1 M HCl.

The samples in all treatments were subjected to 5 min of incubation on an orbital shaker (100 rpm). The samples were then immediately stored in the freezer at − 20 °C before analysis, which was performed within 1 week.

### Chemicals and analysis

The targeted pharmaceuticals were azithromycin, erythromycin, sulfamethoxazole, caffeine, fluconazole, tramadol, trimethoprim, metoprolol, lidocaine, venlafaxine and desvenlafaxine. These compounds were selected based on occurrence and distribution in the aquatic environment, and commercial production and consumption patterns [13]. Individual stocks of the studied compounds were prepared in methanol at a concentration of 1 mg/mL. The working mixture for the spiking experiment was mixed in methanol from these stocks to a concentration of 10 μg/mL. For the chemical analysis, reference standards were purchased from Sigma-Aldrich (Sweden). Isotopically labelled internal standards were purchased from Wellington Laboratories (Canada) and Toronto Research Chemicals (Toronto, Canada). All analytical standards were of high analytical grade (> 95%). Spiking was performed by adding 10 ng of internal standard to 1 mL of filtered sample. The samples were analyzed using a DIONEX UltiMate 3000 ultra-high pressure liquid chromatography (UPLC) system (Thermo Scientific, Waltham, MA, USA) coupled to a triple quadrupole mass spectrometer (MS/MS) (TSQ QUANTIVA, Thermo SCIENTIFIC, Waltham, MA, USA). An Acquity UPLC BEH-C18 column (Waters, 100 mm × 2.1 i.d., 1.7 µm particle size from Waters Corporation, Manchester, UK) was used. Injection volume was 10 µL for all samples. Heated electrospray ionization (H-ESI) was used to ionize the target compound. The spray voltage was set to static: positive ion (V) 3500. Nitrogen (purity > 99.999%) was used as a sheath gas (50 arbitrary units), auxiliary gas (15 arbitrary units) and sweep gas (2 arbitrary units). The vaporizer was heated to 400°C and the capillary to 325°C. The mobile phase consisted of MQ with 5 mM ammonium acetate and acetonitrile. The flow rate was 0.5 mL/min and run time was 15 min.

Xcalibur software (Thermo Fisher Scientific, San Jose, CA, USA) was used for optimizing the instrumental methods and running samples. The data obtained were evaluated using TraceFinderTM 3.3. software (Thermo Fisher).

No target compounds were detected in method blanks and control samples.

### Statistical analysis

All experiments were established with three replicates, and mean and standard deviation for these are reported. The data were analyzed by analysis of variance followed by Fisher’s test. Differences were considered significant at p < 0.05 (Minitab, version 19).

## Results and discussion

### Enzymatic activity

**Fig. 1:**
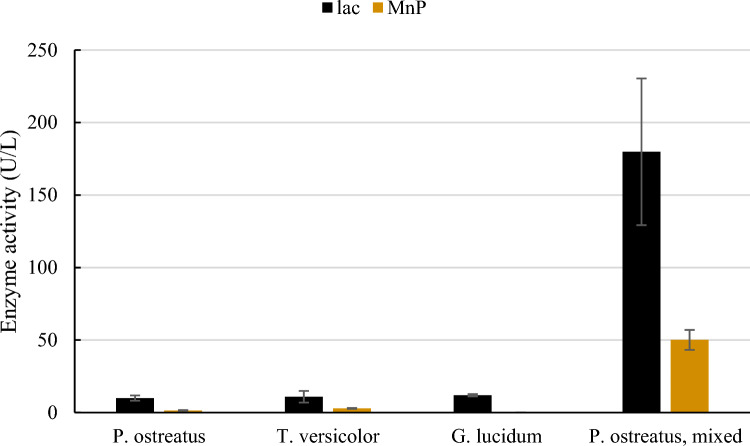
Laccase (lac) activity and manganese peroxidase (MnP) activity in crude enzyme suspensions from birch sawdust colonized with the white-rot fungi *Pleurotus ostreatus*, *Trametes versicolor*, and *Ganoderma lucidum*. The colonized substrates (200 g wet weight) were soaked in 1 L of sterile distilled water for 15 min. For *P. ostreatus*, enzyme activity after brief homogenization (15 s) of the colonized substrate was also determined. Mean ± SD, n = 3 (colour figure online)

White-rot fungi play a natural role in ecosystems as lignin degraders, and there is increasing interest in their use within environmental biotechnology. As mentioned, this is because of their enzyme production, which allows them to take part in degradation of a wide range of persistent compounds [16]. These enzymes also play a role in modification and valorization of lignin into new products [17]. In the present study, laccase and manganese peroxidase activity of three well-known species of white-rot fungi, cultivated under similar conditions, was compared (Fig. 1).

Laccase (lac) activity in crude enzyme suspensions from colonized sawdust was similar for the three white-rot fungi, ranging between 10 and 12 U/L. Laccase activity was significantly higher than manganese peroxidase (MnP) activity, which was detected for *P. ostreatus* and *T. versicolor*, but not for *G. lucidum*. The specific profile of ligninolytic enzymes produced during solid-state fermentation of white-rot fungi is known to be affected by the substrate used [18, 19]. Additionally, laccase activity is commonly reported to exceed the activity of peroxidases in solid-state fermentation on lignocellulosic substrates [20], as also observed in the present study. It should, however, be pointed out that the presence of other ligninolytic enzymes such as lignin peroxidase was not evaluated in the present study. Thus, there might be differences in the ligninolytic enzyme profile between the species which were not detected.

Brief homogenization of sawdust substrate colonized by *P. ostreatus* increased the enzyme activity considerably, with an almost 20-fold increase recorded for laccase and an almost 50-fold increase for manganese peroxidase (Fig. 1). This increase in enzyme activity as a result of homogenization can be explained by several different factors, e.g., physical factors such as break-up of larger strands of mycelium and cellular damage allowing leakage of intracellular enzymes might have an impact. It should be noted that apart from extracellular laccases, intracellular laccases have also been reported in fungi [21]. In addition, disruption of some plant cells can be expected during homogenization, and thereby release of small phenolic compounds, which may act as mediators and contribute to increasing enzyme activity [19, 22]. Also, an increase in oxygen levels in the water suspension after homogenization should be considered an explanation for the increased activity as laccases use oxygen as their terminal oxidant [23]. The pH of the water suspension after 15 min of extraction was 4.8 ± 0.1 for *P. ostreatus* and this pH persisted after mixing. For *T. versicolor* similar pH as for *P. ostreatus* was recorded (4.7 ± 0.2), while the water extracts of sawdust colonized by *G. lucidum* had significantly lower pH (4.3 ± 0.1).

Monitoring of laccase activity in sawdust colonized by *P. ostreatus* over time in the bioreactors indicated that the enzymes were washed out very rapidly. Initial laccase activity in the outlet water in Exps. A and B was 35–40 U/L, but after less than 1 h enzyme activity was below 5 U/L and continued to decrease in both treatments. Thus, no impact of substrate aeration (Exp. B) was observed. Approximately, a total activity of 20 U were recovered from 100 g of the substrate (0.2 U/g substrate) in both treatments.

In Exps. C and D, the flow rate was reduced from 1 to 0.3 L/h and the bioreactors were monitored over a longer period (10 days), in order to assess whether it was possible to achieve fungal growth and thereby increase enzyme activity under these conditions. However, despite that some fungal growth was observed visually laccase activity in the outgoing water was practically negligible in both treatments. Our results suggest that the straightforward approach of using colonized sawdust as filter material is not optimal when high enzymatic activity is the goal.

### Pharmaceutical removal by crude enzyme suspensions produced on sawdust substrate

In a previous study, which had a similar set-up as the present study, laccase activity of 29 U/L was observed to achieve a significant decrease in diclofenac after a short time of exposure [11]. The enzyme activity obtained from colonized sawdust in the present study after 15 min of extraction was lower, in the range 10–12 U/L. The extraction was therefore prolonged to 2 h, through which water suspensions with activity of 43.0 ± 1.1 (lac) and 54.8 ± 2.1 (MnP) U/L were obtained for *P. ostreatus*, 20.4 ± 1.7 (lac) and 6.4 ± 1.5 (MnP) U/L for *T. versicolor*, and 12.1 ± 0.9 (lac) and 4.5 ± 2.1 (MnP) for *G. lucidum* were obtained. Thus, prolonging the extraction time increased the enzyme activity recovered in the crude enzyme suspension. This may relate to the hydrophobicity of the mycelial mats [24], which may have an impact on the diffusion rate of enzymes into the water phase. The pH of the crude enzyme suspensions was not affected by prolonging the extraction, and ranged between 4.4 and 4.7.

**Fig. 2:**
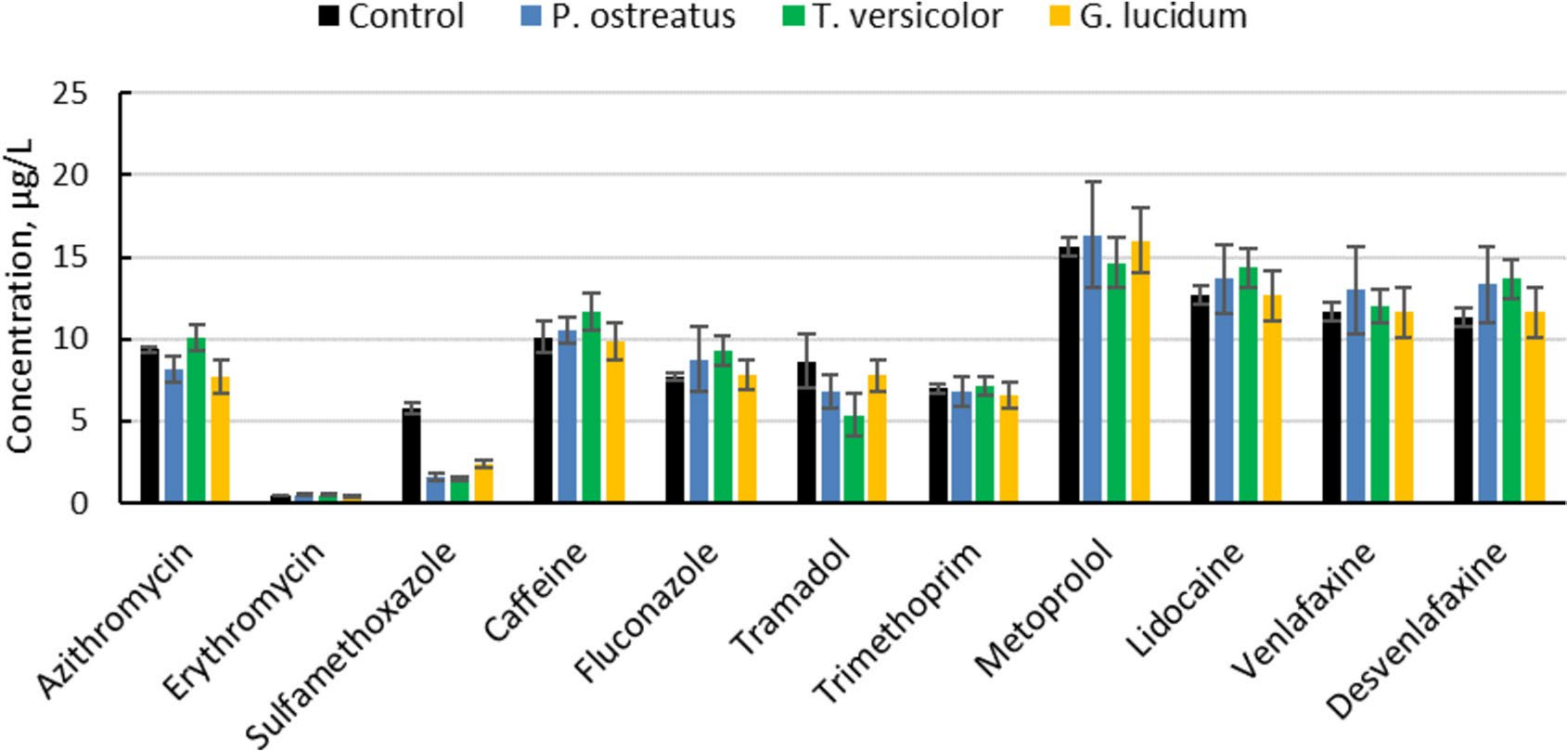
Impact of crude enzyme suspensions of laccase (lac) and manganese peroxidase (MnP) in removal of selected pharmaceuticals from spiked water. Enzyme suspensions based on the fungi *Pleurotus ostreatus* (lac 43.0 ± 1.1, MnP 54.8 ± 2.1 U/L), *Trametes versicolor* (lac 20.4 ± 1.7, MnP 6.4 ± 1.5 U/L), and *Ganoderma lucidum* (lac 12.1 ± 0.9, MnP 4.5 ± 2.1) were produced through soaking sawdust colonized by these white-rot fungi in water. Mean ± SD, n = 3 (colour figure online)

The impact of the crude enzyme suspensions in removing the selected pharmaceuticals was observed to be low (Fig. 2). No impact on total concentration of pharmaceuticals in the water suspension was observed in any treatment. Total sum of pharmaceuticals was 99.5 ± 4.1 µg/L in the autoclaved control, while treatments had a total sum of 102 ± 14.2 (*P. ostreatus*), 101 ± 7.1 (*T. versicolor*) and 94.6 ± 11.5 (*G. lucidum*) µg/L. Compared with the autoclaved control without enzyme activity, a significant decrease in concentration was observed for the compound sulfamethoxazole in all treatments. This finding is well in line with results reported in a recent study, where high removal rates of several sulfonamides were achieved when both artificially contaminated water and real wastewater were exposed to substrate colonized by *P. ostreatus* [25]. As noted in that study, other studies in the recent decade have also confirmed the sensitivity of sulfonamides to laccases [26–30]. In the study by Alharbi et al. [26], a considerable concentration of sulfamethoxazole, 5 mg/L, was exposed to commercial laccase from *T. versicolor* (430–460 U/L) and the effluent was observed to be non-toxic. The study by Chang et al. [27] had a similar approach as the present study and tested extracts of mushroom substrate for removal of three sulfonamides, including sulfamethoxazole. Also, in that study, considerably higher concentrations of the antibiotic compound were used compared to the present study. However, their result shows that it was possible to remove 80% of sulfamethoxazole after 48 h of treatment. Considering the short exposure time and moderate levels of enzyme activity applied in the present study, it seems safe to conclude that for the sulfonamides, treatment based on white-rot fungi is a realistic opportunity.

In the next experiment, attempts were made to increase enzyme activity in the crude enzyme suspensions of *P. ostreatus*, in order to assess whether higher removal efficiency of the pharmaceuticals could be obtained. After water extraction, a crude enzyme suspension with the activity of 135 ± 5.5 (lac) and 136 ± 9.2 (MnP) U/L was obtained. For the crude enzyme suspension based on brief homogenization of the sawdust substrate, enzyme activity of 278 ± 10.3 (lac) and 140 ± 22.7 (MnP) U/L was recorded. In two additional treatments, crude suspension based on unmixed sawdust and mixed sawdust was diluted to obtain similar enzyme activity (Table 2).

**Table 2:** Removal of 11 target pharmaceuticals (azithromycin, erythromycin, sulfamethoxazole, caffeine, fluconazole, tramadol, trimethoprim, metoprolol, lidocaine, venlafaxine and desvenlafaxine) after 5 min of exposure to crude enzyme suspensions of the white-rot fungus *Pleurotus ostreatus* produced on birch sawdust substrate, in which laccase (lac) and manganese peroxidase (MnP) activity was determined (mean ± SD, n = 3)

Treatment	lac (U/L)	MnP (U/L)	Compounds significantly reduced by treatment
Unmixed sawdust	135 ± 5.5	136 ± 9.2	Sulfamethoxazole (75%), trimethoprim (30%)
Unmixed sawdust, diluted	78.3 ± 4.2	57.1 ± 5.7	Sulfamethoxazole (71%)
Mixed sawdust, high enzyme activity	278 ± 10.3	140 ± 22.7	Sulfamethoxazole (84%), trimethoprim (59%), metoprolol (29%), lidocaine (29%), venlafaxine (29%)
Mixed sawdust, diluted	82.4 ± 2.7	59.8 ± 0.8	Sulfamethoxazole (71%), trimethoprim (37%), metoprolol (27%), venlafaxine (30%)

Compounds that showed significant reductions compared with the control are listed. Relative reduction compared with autoclaved controls without enzyme activity is presented in parenthesis

In this second experiment, the total concentration of pharmaceuticals in the water suspension of the autoclaved control was 125 ± 3.0 µg/L. A significant effect of treatment with the mixed suspension with the highest enzyme activity was observed, giving a total concentration of pharmaceuticals of 85.6 ± 20.6 µg/L. No differences were observed for the other treatments, which had total concentrations of 102 ± 19.7 (unmixed), 116 ± 11.7 (unmixed and diluted) and 94.4 ± 26.8 (mixed and diluted) µg/L. Compared with the autoclaved control without enzyme activity, a significant decrease was observed for five pharmaceuticals (sulfamethoxazole, trimethoprim, metoprolol, lidocaine, venlafaxine) in the crude enzyme suspension with the highest activity.

**Fig. 3:**
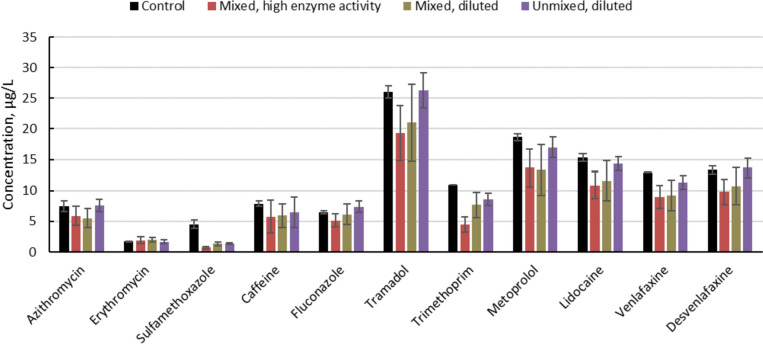
Removal from spiked water of a mixture of different pharmaceuticals exposed to crude enzyme extractions of the white-rot fungus *Pleurotus ostreatus*. The enzyme suspensions were produced on birch sawdust substrate and either briefly mixed or left undisturbed (unmixed). Two of the enzyme suspensions were diluted (“mixed, diluted” and “unmixed, diluted”) to obtain similar activity of the ligninolytic enzymes laccase and manganese peroxidase. Pharmaceutical concentrations in the suspensions after a short time of exposure were compared to those in autoclaved controls without enzyme activity. Mean ± SD, n = 3 (colour figure online)

For the crude enzyme suspension based on whole sawdust, only two pharmaceuticals (sulfamethoxazole and trimethoprim) were significantly reduced compared with the control (Table 2). When enzyme activity was further reduced by diluting the suspension, only sulfamethoxazole was significantly reduced, which is in line with the results presented in Fig. 2. It is interesting to note that the diluted enzyme suspensions, based either on slightly homogenized sawdust or on whole sawdust, with similar enzymatic activity differed somewhat in the outcome. The crude enzyme suspension based on slightly homogenized sawdust performed better, with a significant reduction in four compounds, compared with only one for the other diluted enzyme suspensions (Table 2). For the pharmaceuticals that were not significantly removed, there was trend for the concentrations to decrease to a greater extent in the slightly homogenized suspension compared with the diluted and unmixed suspension (Fig. 3).

The results confirm sulfamethoxazole as highly sensitive to degradation by laccases. However, apart from this compound, comparison of removal efficiency obtained in different studies in relation to the enzyme activity applied is difficult. One main reason for this is the wide range of assays and conditions applied in different studies to measure the enzyme activity of white-rot fungi, as thoroughly discussed by Agrawal and Verma [31] for the case of laccase. Our results suggest that with a short exposure time, enzyme activity is highly relevant for the removal efficiency even though the target pharmaceuticals are present in very low concentrations. Increased enzyme activity was obtained in this study following brief homogenization of the colonized sawdust, but this step will increase the workload when applying the treatment. Benefits of homogenization were also observed when applying suspensions with similar enzyme activity, potentially due to increased presence of mediators as discussed above. Increased presence of intracellular or mycelium-associated enzymes within the cytochrome P450 system, which are suggested to be involved in white-rot fungi-mediated degradation [32–34], may also play a role. However, from an applied perspective, it is clear that a considerable amount of research and technological development is needed before a treatment based on in situ production of ligninolytic enzymes is a realistic opportunity at the WWTP. In this context, it should also be pointed out that the focus of the present study was target analysis of widespread organic pollutants where only parent compounds were analyzed. The enzymatic degradation of pharmaceuticals observed in the present study raises the issue of degradation products. Thus, parallel with the development of systems for in situ production of ligninolytic enzymes, the aspect of degradation products needs to be further studied.

## Conclusions

Sawdust colonized by three common white-rot fungi (*P. ostreatus*, *T. versicolor*, and *G. lucidum*) was used for extraction of ligninolytic enzymes in water suspension. The crude enzyme suspensions obtained were evaluated for degradation of azithromycin, erythromycin, sulfamethoxazole, caffeine, fluconazole, tramadol, trimethoprim, metoprolol, lidocaine, venlafaxine and desvenlafaxine. Significant removal of sulfamethoxazole was observed for all treatments. Further tests using crude enzyme suspensions of *P. ostreatus* showed that degradation of a wider range of pharmaceuticals could be achieved by increasing the enzyme activity. Brief homogenization of the colonized sawdust substrate was also observed to be favorable, resulting in significant reductions in sulfamethoxazole, trimethoprim, metoprolol, lidocaine and venlafaxine after a short exposure time (5 min). Monitoring of enzymatic activity over time at the outlet of bioreactors packed with sawdust colonized with *P. ostreatus* revealed a very rapid decrease in enzyme activity. Thus, the straightforward approach of using colonized sawdust as filter material is not optimal when high enzymatic activity is the goal.

## Data Availability

The data will be made available upon request.
